# Suppression of long noncoding RNA LINC00324 restricts cell proliferation and invasion of papillary thyroid carcinoma through downregulation of TRIM29 via upregulating microRNA-195-5p

**DOI:** 10.18632/aging.202219

**Published:** 2020-12-14

**Authors:** Jinkai Xu, Zongyu Li, Qinghua Su, Jun Zhao, Jiancang Ma

**Affiliations:** 1 Department of General Surgery, The Second Affiliated Hospital of Xi'an Jiaotong University, Xi'an 710004, Shaanxi, China

**Keywords:** LINC00324, miR-195-5p, papillary thyroid carcinoma, TRIM29

## Abstract

Long noncoding RNAs (lncRNAs) are identified as novel regulators of carcinogenesis. To date, the precise functions of lncRNAs in papillary thyroid carcinoma (PTC) remains poorly understood. The purposes of this work were to explore the potential relevance of lncRNA 00324 (LINC00324) in PTC. Levels of LINC00324 were markedly up-regulated in PTC. Silencing of LINC00324 significantly repressed the proliferation and invasion of PTC cells. LINC00324 was documented as a sponge of microRNA-195-5p (miR-195-5p). Decreased levels of miR-195-5p were detected in PTC. The up-regulation of miR-195-5p suppressed PTC cellular proliferation and invasion. Suppression of miR-195-5p partially reversed the LINC00324-knockdown-mediated effects in PTC cells. We identified tripartite motif-containing 29 (TRIM29) as a target gene of miR-195-5p. TRIM29 overexpression partially reversed the LINC00324-knockdown- or miR-195-5p-overexpression-mediated effects in PTC cells. In short, this work demonstrates that LINC00324 knockdown inhibits the proliferation and invasion of PTC cells by decreasing TRIM29 expression via up-regulating miR-195-5p expression.

## INTRODUCTION

Thyroid carcinoma is one of the most prevalent endocrine malignancies, and the incidence and mortality of thyroid carcinoma have increased in the last few years [1]. Papillary thyroid carcinoma (PTC) is the most frequent subtype of thyroid carcinoma, comprising approximately 80–85% of primary thyroid carcinomas [2]. On account of the advances in tumor therapy, the 5-year survival rates for PTC patients have prominently improved in recent years [3]. However, a portion of PTC patients may develop recurrent disease and distant metastases that dedifferentiate into deadlier thyroid carcinoma. PTC is a heterogeneous disease with a complicated pathogenesis that frequently involves various genetic alterations [4]. Thus, it is imperative to better understand the molecular events the molecular events occurring during the malignant progression of PTC is imperative, as this knowledge will help to improve PTC diagnosis and treatment.

Long noncoding RNAs (lncRNAs) are a newly identified subtype of noncoding RNAs that have attracted extensive attention [5]. LncRNAs are implicated in a broad-spectrum of cellular activities, such as transcription, translation, protein synthesis, and cellular signaling cascades [6, 7]. Interestingly, lncRNAs often function as competing endogenous RNAs (ceRNAs) that act as natural molecular sponges for miRNAs [8]. Notably, lncRNA dysregulation is implicated in cancers [9]. LncRNAs are dysregulated in PTC and, thus, may serve as prospective novel therapeutic targets as well as diagnostic and prognostic biomarkers [10]. In recent years, increasing studies have aimed to identify the potential lncRNAs involved in PTC [11–13]. However, the precise regulatory mechanism of lncRNAs that underlies PTC pathogenesis remains largely unknown.

It is well-known tat miRNAs are a group of noncoding RNAs which adjust gene expression at the post-transcriptional level [14, 15]. Compared to lncRNAs, miRNAs are shorter in length, composed of approximately 23 nucleotides, and able to bind to the 3′-untranslated region (3′-UTR) of target mRNA, which causes inhibitory effect on gene expression [16]. miRNAs participate in diverse physiological and pathological processes via affecting multiple cellular biological processes [17, 18]. Notably, a variety of miRNAs are found aberrantly expressed in PTC and exert a tumor-promotion or tumor-inhibition role [19, 20]. To date, the regulatory mechanisms of miRNA dysregulation in tumorigenesis remain unclear. Inspiringly, a recent study demonstrated that miRNAs are regulated by ceRNAs, such as lncRNAs [21]. Therefore, identification of a lncRNA-miRNA-mRNA axis in PTC may provide a critical foundation for better understanding the PTC molecular pathogenesis.

Tripartite motif 29 (TRIM29) exerts a crucial function in carcinogenesis [22]. High levels of TRIM29 expression are frequently detected in various types of malignancy, which are associated with advanced tumor grade and reduced survival rate [23]. TRIM29 accelerates the progression of tumors by participating in a variety of signaling pathways [24–27]. Therefore, TRIM29 has been proposed as a possible target for anticancer therapeutic development.

LINC00324 is dysregulated in a number of cancers and exerts tumor-promoting functions [28–32]. To date, the detailed relevance of LINC00324 in PTC has not been fully understood. This work was designed to determine the possible relationship between LINC00324 and PTC.

## RESULTS

### LINC00324 is highly expressed in PTC tissue and cell lines

**Figure 1:**
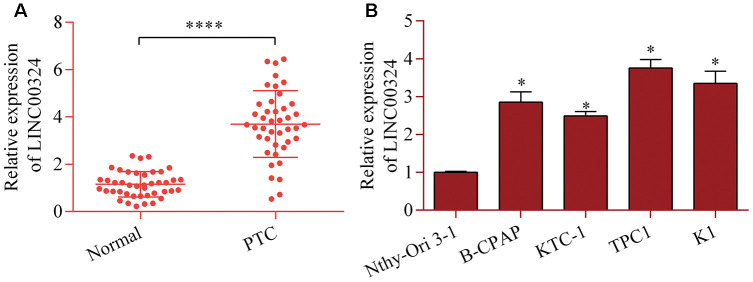
**Increased LINC00324 expression in PTC.** (**A**) Level of LINC00324 in 42 pairs of PTC tissue and normal tissue was measured via RT-qPCR. ****p < 0.0001 versus normal. (**B**) Level of LINC00324 in PTC cell lines (B-CPAP, KTC-1, TPC1, and K1) and Nthy-Ori 3-1 cells was assessed via RT-qPCR. *p < 0.05 versus Nthy-Ori 3-1.

To determine the possible role of LINC00324 in PTC, levels of LINC00324 in PTC tissue were examined using RT-qPCR. LINC00324 levels were markedly up-regulated in PTC tissue relative to adjacent normal tissue (Figure 1A). Additionally, our data further showed significant increases in LINC00324 expression in PTC cell lines (Figure 1B). These results indicate a possible association between LIN00324 and PTC progression.

### LINC00324 knockdown restricts proliferation and invasion of PTC cells

**Figure 2:**
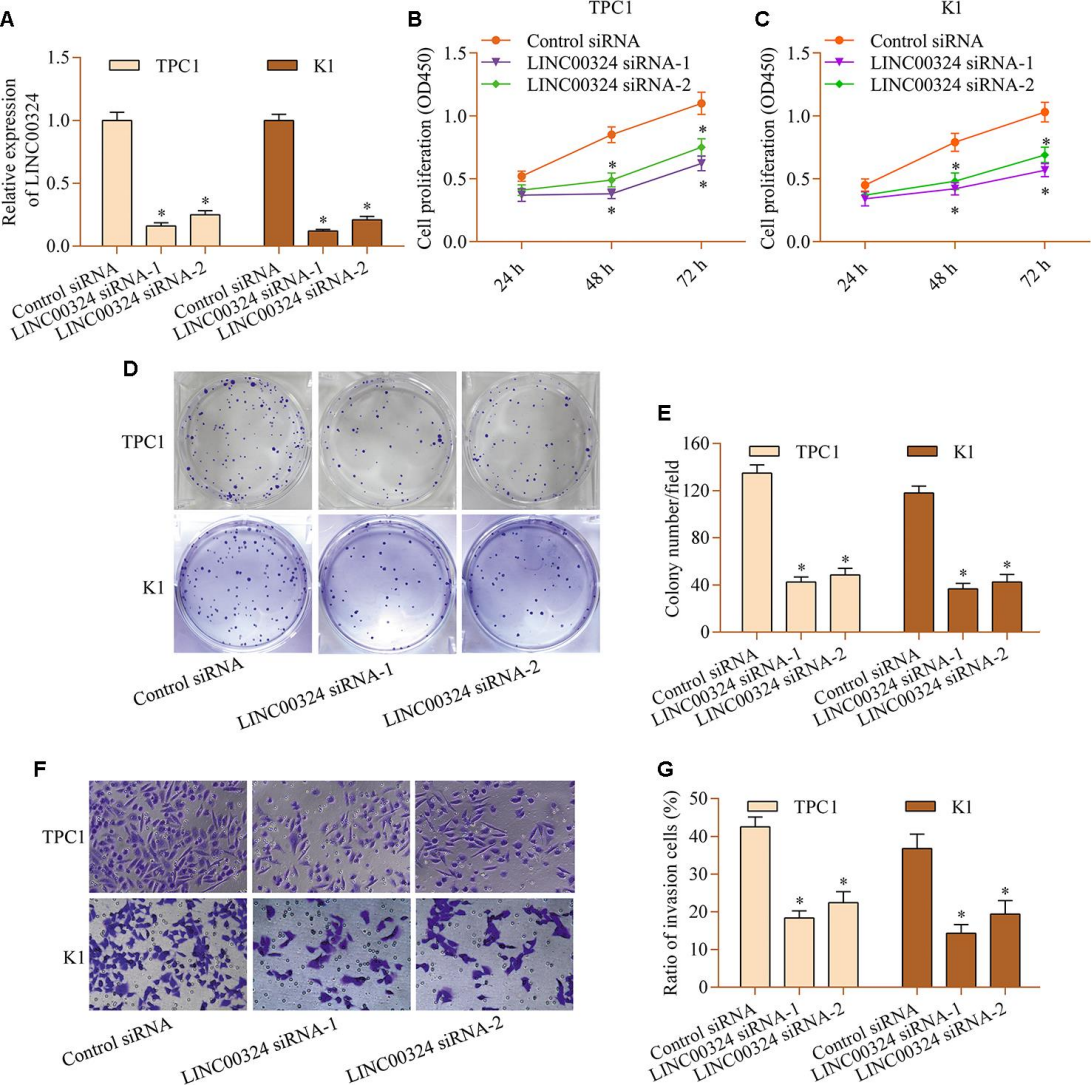
**LINC00324 knockdown restricts the proliferation and invasion of PTC cells *in vitro*.** TPC1 and K1 cells were transfected with LINC00324 siRNA or control siRNA for 48 h, and (**A**) knockdown of LINC00324 was confirmed via RT-qPCR. (**B**, **C**) Effect of LINC00324 knockdown on cellular proliferation was assessed via a CCK-8 assay. (**D**, **E**) Effect of LINC00324 knockdown on clonogenic growth was detected via a colony formation assay. (**F**, **G**) Effect of LINC00324 silencing on invasive potential was determined via a transwell invasion assay. *p < 0.05 versus control siRNA.

To determine the role of LINC00324 in PTC, the loss-of-function experiments for LINC00324 was carried out via using siRNA-mediated gene silencing. Transfection with siRNAs that specifically target LINC00324 markedly depleted LINC00324 expression (Figure 2A). LINC00324 knockdown markedly decreased proliferation of TPC1 and K1 cells (Figure 2B and 2C). The colony-forming capabilities of TPC1 and K1 cells was down-regulated by LINC00324 knockdown (Figure 2D and 2E). Further, LINC00324 silencing also weakened invasive ability of TPC1 and K1 cells (Figure 2F and 2G). The data suggest that LINC00324 inhibition produces antitumor effects in PTC cells.

### LINC00324 acts as a sponge of miR-195-5p

**Figure 3:**
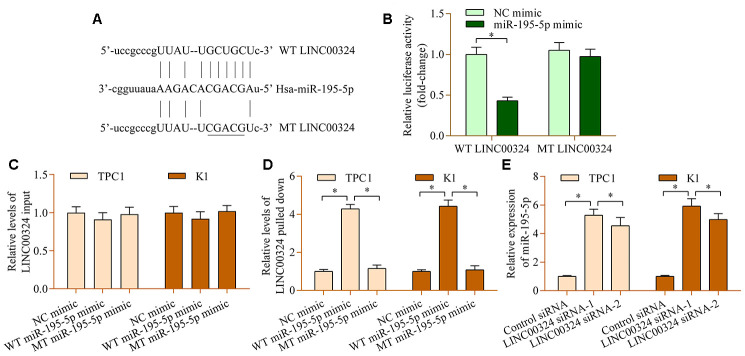
**miR-195-5p interacts with LINC00324 in PTC cells.** (**A**) Sequence alignment between miR-195-5p and WT or MT LINC00324. (**B**) A dual-luciferase reporter assay was carried out using 293T cells cotransfected with WT or MT LINC00324 reporter vectors and miR-195-5p mimic. Relative levels of LINC00324 (**C**) input and (**D**) pulled-down in RNA pull-down assay. An RNA pull-down assay was carried out using biotinylated WT or MT miR-195-5p mimic in TPC1 and K1 cells. LINC00324 expression in RNA precipitates was examined by RT-qPCR. (**E**) Levels of lINC00324 in TPC1 and K1 cells transfected with LINC00324 siRNA or control siRNA were assessed via RT-qPCR. *p < 0.05.

Next, the potential miRNAs that are sponged by LINC00324 were explored by using the online bioinformatic tool ENCORI. Among these potential targets, miR-195-5p, a tumor-inhibition miRNA in PTC [33], attracted our interest. To test whether miR-195-5p binds to LINC00324 at the predicted site, a dual-luciferase reporter assay was carried out. LINC00324 cDNA fragments harboring a miR-195-5p wild-type (WT) or mutant (MT) site were inserted into reporter plasmids to construct the LINC00324 reporter (Figure 3A). Our data demonstrated that up-regulation of miR-195-5p down-regulated luciferase activity of the WT LINC00324 reporter, whereas miR-195-5p had no obvious effect on the MT LINC00324 reporter (Figure 3B). Moreover, the results of RNA pull-down showed that LINC00324 was abundantly existed in miR-195-5p mimic-precipitated RNA transcripts (Figure 3C, 3D). Notably, LINC00324 depletion increased the levels of miR-195-5p (Figure 3E). Collectively, the findings confirm that LINC00324 acts as a sponge of miR-195-5p.

### miR-195-5p contributes to LINC00324-mediated effects in PTC cells

**Figure 4:**
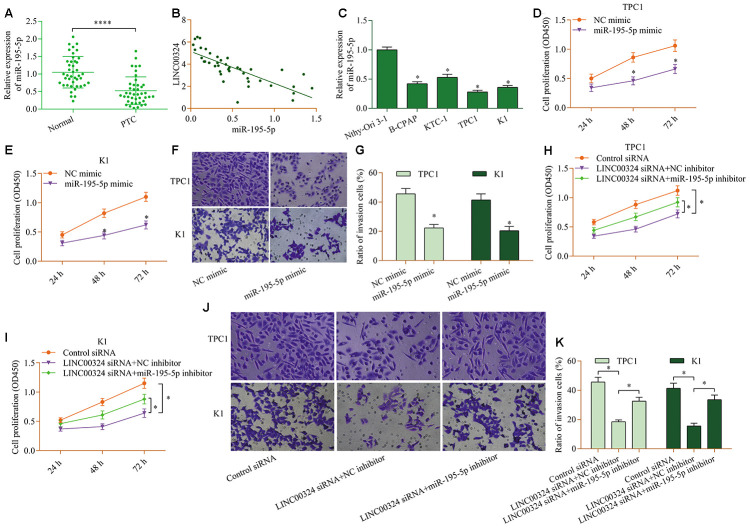
**miR-195-5p contributes to LINC00324-mediated effects in PTC cells.** (**A**) Levels of miR-195-5p in PTC tissue and normal tissue were examined via RT-qPCR. ****p < 0.0001 versus normal. (**B**) miR-195-5p was inversely associated with LINC00324 in PTC tissue (r=-0.8715, p<0.0001). (**C**) Levels of miR-195-5p in PTC cell lines (B-CPAP, KTC-1, TPC1, and K1) and Nthy-Ori 3-1 cells were measured via RT-qPCR. *p < 0.05 versus Nthy-Ori 3-1. (**D**, **E**)Effect of miR-195-5p overexpression on cellular proliferation of TPC1 and K1 cells was assessed via a CCK-8 assay. (**F**, **G**) Effect of miR-195-5p overexpression on the invasive potential of TPC1 and K1 cells was evaluated by a transwell invasion assay. *p < 0.05 versus NC mimic. TPC1 and K1 cells were transfected with LINC00324 siRNA and miR-195-5p inhibitor for 48 h, and (**H**, **I**) cellular proliferation was evaluated via a CCK-8 assay, and (**J**, **K**) cellular invasion was monitored via a transwell invasion assay. *p < 0.05.

To determine the role of miR-195-5p in PTC, we analyzed the levels of miR-195-5p in PTC. The results demonstrated remarkable decreases in miR-195-5p levels in PTC tissue (Figure 4A). Furthermore, miR-195-5p was inversely associated with LINC00324 (Figure 4B). Additionally, low level of miR-195-5p was also detected in PTC cell lines (Figure 4C). Functional experiments revealed that miR-195-5p overexpression prominently reduced the proliferation (Figure 4D and 4E) and invasion (Figure 4F and 4G) of PTC cells lines. Notably, suppression of miR-195-5p markedly abrogated LINC00324-depletion-induced effects on PTC cell proliferation (Figure 4H and 4I) and invasion (Figure 4J and 4K). Altogether, these data confirm a cancer-inhibition role for miR-195-5p in PTC and suggest that it participates in modulation of LINC00324-mediated effects in PTC.

### miR-195-5p negatively modulates TRIM29

**Figure 5:**
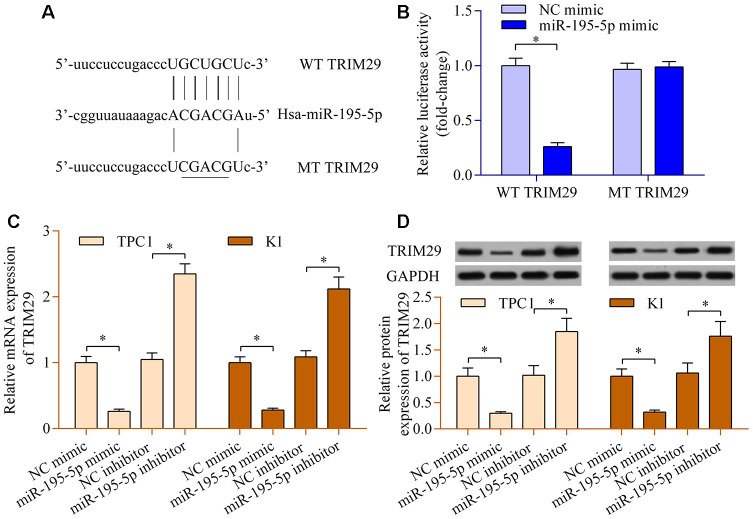
**miR-195-5p negatively modulates TRIM29 expression.** (**A**) Sequence alignment between miR-195-5p and WT or MT TRIM29 3′-UTR. (**B**) A dual-luciferase reporter assay was carried out using 293T cells cotransfected with WT or MT TRIM29 3′-UTR reporter vectors and miR-195-5p mimic. TPC1 and K1 cells were transfected with miR-195-5p mimic, miR-195 inhibitor, or NC controls for 48 h. (**C**) Levels of TRIM29 mRNA were determined via RT-qPCR. (**D**) Levels of TRIM29 protein were evaluated via western blot. *p < 0.05.

To further reveal the underlying mechanisms of the LINC00324/miR-195-5p axis in PTC, the potential downstream target of LINC00324/miR-195-5p axis was further explored. Bioinformatic analysis predicted that TRIM29, a well-known oncogene [23], contains a putative miR-195-5p binding site at 3′-UTR (Figure 5A). The overexpression of miR-195-5p restrained the production of luciferase activity of WT TRIM29 3′-UTR reporter, while it had no significant impact on MT TRIM29 3′-UTR reporter (Figure 5B). In addition, miR-195-5p overexpression observably decreased TRIM29 expression, whereas suppression of miR-195-5p up-regulated TRIM29 expression (Figure 5C and 5D). The data hint that miR-195-5p negatively modulates the expression of TRIM29 in PTC.

### TRIM29 overexpression reverses the miR-195-5p-overexpression- or LINC00324-knockdown-mediated effects in PTC cells

**Figure 6:**
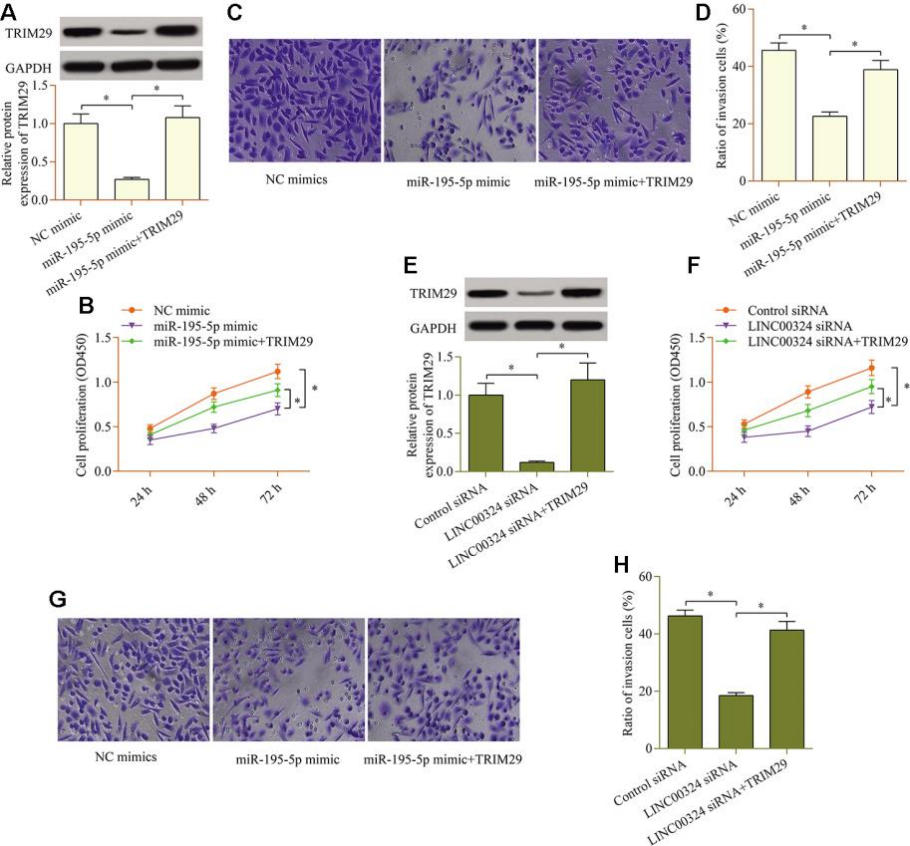
**TRIM29 overexpression reverses the miR-195-5p-overexpression- or LINC00324-knockdown-mediated effects in PTC cells.** miR-195-5p mimic and pcDNA3.1/TRIM29 vectors were cotransfected into TPC1 cells, and (**A**) protein levels of TRIM29 were examined via western blot. (**B**) Effect of TRIM29 overexpression on miR-195-5p-mediated cellular proliferation was determined via a CCK-8 assay. (**C**, **D**) Effect of TRIM29 overexpression on miR-195-5p-mediated cellular invasion was evaluated via a transwell invasion assay. LINC00324 siRNA and pcDNA3.1/TRIM29 vectors were cotransfected into TPC1 cells, and (**E**) protein levels of TRIM29 was examined via western blot. (**F**) Effect of TRIM29 overexpression on LINC00324 knockdown-mediated cellular proliferation was measured via a CCK-8 assay. (**G**, **H**) Effect of TRIM29 overexpression on LINC00324 knockdown-mediated cellular invasion was monitored via a transwell invasion assay. *p < 0.05.

To validate whether TRIM29 contributes to LINC00324/miR-195-5p axis-mediated effects in PTC cells, a rescue assay was carried out. Transfecting a TRIM29 expression plasmid markedly up-regulated the levels of TRIM29 in cells overexpressing miR-195-5p (Figure 6A). TRIM29 overexpression observably abolished the repressive impact of miR-195-5p overexpression on cellular proliferation (Figure 6B) and invasion (Figure 6C, 6D). Similarly, the LINC00324-knockdown-mediated effects were also partially reversed by TRIM29 overexpression (Figure 6E–6H). Collectively, these findings validate that TRIM29 acts as a functional target of the LINC00324/miR-195-5p axis.

## DISCUSSION

This work for the first time identified a vital role of LINC00324 in PTC. Our data showed significant increases in LINC00324 levels in PTC tissues and cell lines, and demonstrated that LINC00324 depletion produced tumor-inhibition effects in PTC cells *in vitro*. It is noteworthy that lncRNAs function as ceRNAs via sequestering miRNAs. The sponged miRNAs lose their ability to degrade or repress the translation of their downstream target mRNAs. Our results further reveal that LINC00324 exerts its function in PTC by regulating TRIM29 via sequestering miR-195-5p. This work underscores a vital role of LINC00324/miR-195-5p/TRIM29 axis in PTC progression.

LINC00324 is involved in regulating cell survival by sequestering miR-615-5p to regulate the expression of B-cell translocation gene 2 [34]. Notably, LINC00324 dysregulation is involved in tumorigenesis. LINC00324 levels are markedly up-regulated in lung adenocarcinoma, and LINC00324 overexpression promotes the progression of lung adenocarcinoma through upregulation of Akt1 via sequestering miR-615-5p [29]. LINC00324 overexpression in gastric cancer correlates with larger tumor sizes, lymph node metastasis, and low survival rate [28]. LINC00324 knockdown impedes the growth of gastric cancer via down-regulating FAM83B via combining with RNA-binding protein human antigen R [28]. Up-regulation of LINC00324 has been detected in osteosarcoma and LINC00324 has been shown to accelerate the proliferation and migration of osteosarcoma via stabilizing WD repeat-containing protein 66 [32]. In colorectal cancer, knockdown of LINC00324 exerts a tumor-inhibiting role via sequestering miR-214-3p [35]. These studies propose a potential cancer-promoting function of LINC00324. Moreover, the cancer-promoting function of LINC00324 is evidenced in other cancers, including liver cancer, retinoblastoma, and lung cancer [30, 31, 36]. In this work, our results elucidated that LINC00324 levels were up-regulated in PTC and LINC00324 knockdown produced antitumor effects in PTC cells. Intriguingly, the findings of our work are in line with a recent study reporting that LINC00324 expression is elevated in PTC, and silencing of LINC00324 exhibits a tumor-inhibition role in PTC cells [37]. Therefore, this work confirms a cancer-promoting function for LINC00324 in PTC and suggests this lncRNA as a possible target for PTC treatment.

miR-195-5p is documented as a tumor-inhibition miRNA in numerous tumors [38, 39]. Interestingly, the relevance of miR-195-5p in PTC has been reported by several studies. Levels of miR-195-5p are found down-regulated in PTC [40]. Overexpression of miR-195-5p restrained the proliferation and invasion of PTC cells via downregulation of CCND1, FGF2, and Raf1 [33, 41]. Consistent with these findings, our work verifies the down-regulation of miR-195-5p in PTC. Moreover, this work identified TRIM29 as a novel target of miR-195-5p. TRIM29 overexpression significantly reversed the miR-195-5p-mediated effects in PTC cells. In our previous study [25], we reported high levels of TRIM29 in PTC, and that TRIM29 depletion suppressed the proliferative and invasive capabilities of PTC cells; findings that suggest a cancer-promotion function of TRIM29 in thyroid carcinoma. Therefore, the low levels of miR-195-5p in PTC may induce high levels of TRIMP29 that consequently trigger tumor progression.

Although the tumor-inhibition function of miR-195-5p in PTC has been well established, many questions remain concerning the mechanism of miR-195-5p dysregulation in cancers. Interestingly, recent evidence has documented that miR-195-5p is controlled by ceRNAs, including lncRNAs [42, 43]. Indeed, when sequestered by lncRNAs, miR-195-5p loses its ability to degrade or repress the translation of its downstream target mRNAs [44, 45]. Our work identifies LINC00324 as a sponge of miR-195-5p. Our results demonstrated that LINC00324 directly interacted with miR-195-5p, and that LINC00324 knockdown significantly increased miR-195-5p level in PTC. Furthermore, inhibition of miR-195-5p markedly reversed LINC00324 knockdown-induced tumor-inhibiting effect. Additionally, LINC00324 knockdown down-regulated the levels of TRIM29 in PTC cells, and TRIM29 overexpression markedly abolished LINC00324-knockdown- or miR-195-5p-overexpression-mediated cancer-inhibiting effects. In short, this work indicates that the LINC00324/miR-195-5p/TRIM29 axis may represent a novel regulatory mechanism for PTC progression.

Some limitations of this work need to be noted. Although our study revealed a vital role of LINC00324/miR-195-5p/TRIM29 in the progression of PTC, questions remain concerning the relevance of LINC00324/miR-195-5p/TRIM29 axis in other subtypes of thyroid carcinoma. Therefore, additional studies are needed to assess whether the LINC00324/miR-195-5p/TRIM29 axis is restricted to PTC or if it extends to other subtypes of thyroid cancer.

**Figure 7:**
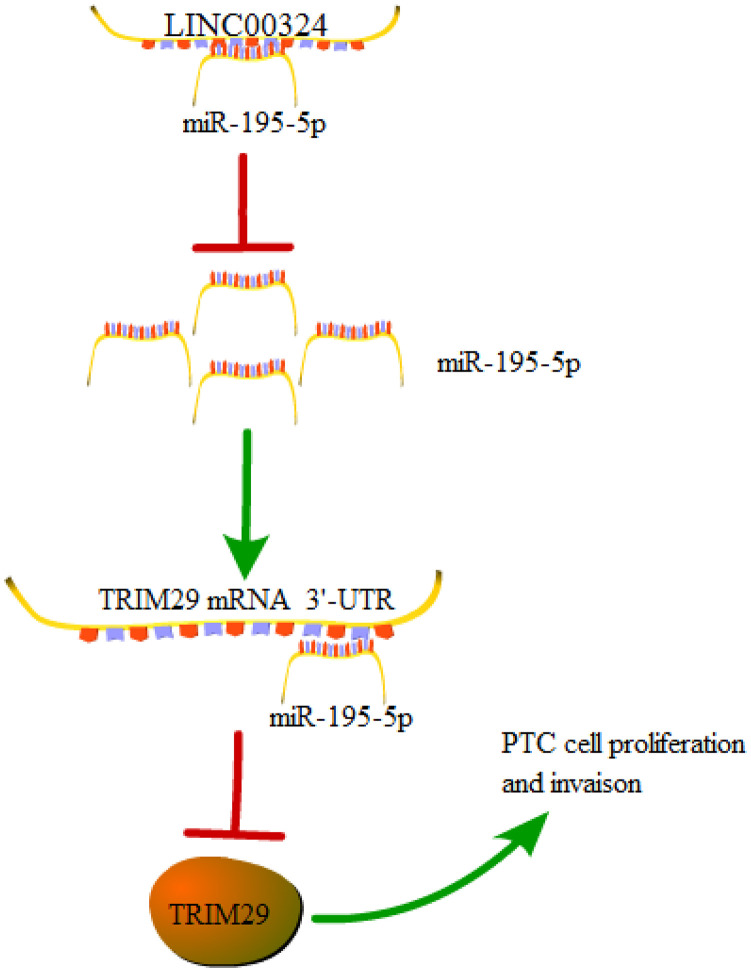
**A graphical model of the LINC00324/miR-195-5p/TRIM29 axis in PTC.**

In conclusion, our findings elucidate that LINC00324 inhibition restrains the proliferative and invasive abilities of PTC cells through down-regulation of TRIM29 via increasing miR-195-5p. These findings highlight a crucial function for the LINC00324/miR-195-5p/TRIM29 axis in PTC progression (Figure 7). This work may offer novel insights into understanding PTC progression and help to exploit new and effective anticancer therapies for PTC patients.

## MATERIALS AND METHODS

### PTC specimens

Forty-two pairs of PTC tissue and adjacent non-cancerous tissue were provided by the Department of General Surgery of The Second Affiliated Hospital of Xi'an Jiaotong University. Written informed consents from participants were acquired prior to tissue donation for study purposes. The use and study of clinical specimens were approved by the Ethics Committee of Xi'an Jiaotong University. Experimental protocols were performed in compliance with the guidelines and principles of the Declaration of Helsinki.

### Cell culture

Human PTC cell lines (B-CPAP, KTC-1, TPC1, and K1), a normal thyroid cell line (Nthy-Ori 3-1), and 293T cells were used in this study. B-CPAP, KTC-1, and 293T cell lines were provided by Stem Cell Bank, Chinese Academy of Sciences (Shanghai, China). TPC1, K1, and Nthy-Ori 3-1 cell lines were obtained from BeNa Culture Collection (BNCC, Kunshan, China). Cells were cultivated in accordance with the manufacturer’s standard culture methods. Generally, B-CPAP, KTC-1, and Nthy-Ori 3-1 cell lines were grown in RPMI Medium 1640 harboring 10% fetal bovine serum (FBS), while 293T, TPC1, and K1 cell lines were maintained in Dulbecco Modified Eagle Medium harboring 10% FBS. All cell lines were maintained in an atmosphere of air containing 5% CO_2_ at 37° C.

### RNA extraction and RT-qPCR analysis

RNeasy Mini Kit (Qiagen, Dusseldorf, Germany) was applied to extract total RNA as per the manufacturer’s protocols. TaqMan Reverse Transcription Reagents Kit (Invitrogen; Thermo Fisher Scientific, Waltham, MA, USA) was utilized to reversely transcribe total RNA into cDNA. Amplification of cDNA was achieved via PCR using Power SYBR Green PCR Master Mix (Applied Biosystems; Thermo Fisher Scientific). GAPDH was utilized as an internal referee for normalizing mRNA and lncRNA expression. Total miRNA was isolated via the mirVana miRNA Isolation Kit (Invitrogen) and reversely transcribed into cDNA via TaqMan MicroRNA Reverse Transcription Kit (Applied Biosystems) as per the manufacturers’ manuals. RT-qPCR for detecting miR-195-5p levels was carried out with TaqMan Fast Advanced Master Mix (Applied Biosystems). U6 was applied as an internal referee for normalizing miR-195-5p expression. The primer sequences for gene amplification in RT-qPCR were as follows: LINC00324 sense, 5’-TGTGGATGACAGTGTTCGGG-3’ and antisense, 5’-ACGCTGACCAGAAACCGTAG-3’; TRIM29 sense, 5’-ATGCTTGGTGGTCACTTTGG-3’ and antisense, 5’-GCACTTCCCTTACCAGCATAG-3’; GAPDH sense, 5’-GAAGGTGAAGGTCGGAGTC-3’ and antisense, 5’-GAAGATGGTGATGGGATTTC-3’; miR-195-5p sense, 5’-CGCAGTAGCAGCACAGA-3’ and antisense, 5’-TCCAGTTTTTTTTTTTTTTTGCCA-3’; U6 sense, 5’-CTCGCTTCGGCAGCACA-3’ and antisense, 5’-AACGCTTCACGAATTTGCGT-3’.

### Cell transfection

siRNA sequences that specifically targeted LINC00324 (siRNA-1: 5’-CGUUUAUCAGUGGUUGGAA-3’ and siRNA-2: 5’GUGUCAAGAUCCCAGGUUA-3’) were synthesized by Genepharma (Shanghai, China). The miRNA mimic (5’- UAGCAGCACAGAAAUAUUGGC-3’), inhibitor (5’-GCCAAUAUUUCUGUGCUGCUA-3’), and negative control (NC: 5’-UUCUCCGAACGUGUCACGUTT-3’) for miR-195-5p were purchased from RiboBio (Guangzhou, China). The open reading frame of TRIM29 cDNA was subcloned into pcDNA3.1 plasmids to generate pcDNA3.1/TRIM29 constructs. Lipofectamine 3000 Transfection Kit (Thermo Fisher Scientific, Waltham, MA, USA) was utilized to transfect oligonucleotides and/or plasmids into PTC cells complied with the manufacturer’s manuals.

### Cell proliferation assay

Transfected PTC cells were seeded into a 96-well plate and cultured for 48 h. CCK-8 solution (Beyotime, Shanghai, China) were added to cells at 10 μl/well, followed by incubation for 1 h. Optical density (OD) at 450 nm was assessed with a microplate reader (BioTek, Winooski, VT, USA).

### Colony formation assay

Suspensions of transfected PTC cells were seeded in a 6-well tissue culture plate at 500 cells/well. The plates were placed into a cell incubator and cultivated for 14 days at 37° C. To visualize colonies, colonies were fixed by methanol before being dyed with crystal violet. Numbers of colonies were counted via an inverted microscope.

### Transwell invasion assay

BioCoat Matrigel Invasion Chambers (BD Biosciences, San Jose, CA, USA) were applied to determine the invasive capability of PTC cells. Generally, suspensions of transfected PTC cells in serum-free medium were added into the BioCoat Matrigel Invasion Chambers. The lower wells were added with medium that contained 20% FBS. The chambers were placed into a cell incubator for 24 h at 37° C. Subsequently, invaded cells were fixed with methanol and dyed with crystal violet. Numbers of invasive cells were counted via a microscope, and the invasion index was determined by the formula: number of cells invaded through Matrigel insert membrane/number of cells migrated through control insert membrane.

### Bioinformatic analysis

The potential miRNAs sponged by LINC00324 were explored via bioinformatic tool ENCORI: The Encyclopedia of RNA Interactomes (http://starbase.sysu.edu.cn/index.php). The targets of miR-195-5p were explored via bioinformatic tools TargetScan (http://www.targetscan.org/vert_71/) and Targets and Expression via microRNA.org.

### Luciferase reporter assay

The cDNA fragment of LINC00324 and TRIM29 3′-UTR that contained a putative miR-195-5p binding site was subcloned into the pmirGLO dual-luciferase reporter vectors (Promega, Madison, WI, USA). Meanwhile, mutant reporter vectors for LINC00324 or TRIM29 3′-UTR that harbored the mutated miR-195-5p binding site were also constructed. The reporter constructs and miR-195-5p mimics were cotransfected into 293T cells. After being cultivated for 48 h, cells were harvested and lysed to measure the luciferase activity produced by the reporter constructs following the manufacturer’s manuals of Dual-Luciferase Reporter Assay System (Promega).

### RNA pull-down with biotinylated miRNA

Biotin RNA Labeling Mix (Roche Diagnostics, Indianapolis, IN, USA) was applied to label miRNAs with biotin. PTC cells were transfected with biotinylated miRNAs for 48 h. Cells were then harvested and lysed in lysis buffer. The lysates were incubated with Streptavidin-Coated Magnetic Beads (Thermo Fisher Scientific) at 4° C for 3 h. The RNAs precipitated by the beads were isolated and purified using the RNeasy Mini Kit (Qiagen). LINC00324 expression in the precipitates was determined using RT-qPCR.

### Western blot analysis

Equivalent amounts of proteins were added to sodium dodecyl sulfate-polyacrylamide gels, which were separated via electrophoresis. Electro-transfer experiments were performed to transfer the proteins from sodium dodecyl sulfate-polyacrylamide gels to polyvinylidene fluoride (PVDF) membranes (Thermo Fisher Scientific). After being blocked, membranes were incubated with primary antibodies against TRIM29 and GAPDH (Abcam, Cambridge, UK) overnight at 4° C. Membranes were washed and probed with horseradish peroxidase-conjugated secondary antibody (Abcam). Membranes were developed with Pierce ECL Plus Western Blotting Substrate (Thermo Fisher Scientific).

### Statistical analysis

Results are presented as mean ± standard deviation (SD). Statistical analyses were carried out with SPSS version 19.0 software (SPSS Inc., Chicago, IL, USA). Comparisons between two groups were determined via Student’s t-test, while comparisons among multiple groups were assessed via one-way analysis of variance (ANOVA) followed by Tukey’s post-hoc test. Differences with p < 0.05 were deemed to be statistically significant.

## References

[1] Kitahara CM, Sosa JA. The changing incidence of thyroid cancer. Nat Rev Endocrinol. 2016; 12:646–53. 10.1038/nrendo.2016.110 27418023 PMC10311569

[2] LiVolsi VA. Papillary thyroid carcinoma: an update. Mod Pathol. 2011 (Suppl 2); 24:S1–9. 10.1038/modpathol.2010.129 21455196

[3] Siegel RL, Miller KD, Jemal A. Cancer statistics, 2019. CA Cancer J Clin. 2019; 69:7–34. 10.3322/caac.21551 30620402

[4] Omur O, Baran Y. An update on molecular biology of thyroid cancers. Crit Rev Oncol Hematol. 2014; 90:233–52. 10.1016/j.critrevonc.2013.12.007 24405857

[5] Kopp F, Mendell JT. Functional classification and experimental dissection of long noncoding RNAs. Cell. 2018; 172:393–407. 10.1016/j.cell.2018.01.011 29373828 PMC5978744

[6] Wang KC, Chang HY. Molecular mechanisms of long noncoding RNAs. Mol Cell. 2011; 43:904–14. 10.1016/j.molcel.2011.08.018 21925379 PMC3199020

[7] Batista PJ, Chang HY. Long noncoding RNAs: cellular address codes in development and disease. Cell. 2013; 152:1298–307. 10.1016/j.cell.2013.02.012 23498938 PMC3651923

[8] Qi X, Zhang DH, Wu N, Xiao JH, Wang X, Ma W. ceRNA in cancer: possible functions and clinical implications. J Med Genet. 2015; 52:710–18. 10.1136/jmedgenet-2015-103334 26358722

[9] Bhan A, Soleimani M, Mandal SS. Long noncoding RNA and cancer: a new paradigm. Cancer Res. 2017; 77:3965–81. 10.1158/0008-5472.CAN-16-2634 28701486 PMC8330958

[10] Murugan AK, Munirajan AK, Alzahrani AS. Long noncoding RNAs: emerging players in thyroid cancer pathogenesis. Endocr Relat Cancer. 2018; 25:R59–82. 10.1530/ERC-17-0188 29146581

[11] Jiang L, Wu Z, Meng X, Chu X, Huang H, Xu C. LncRNA HOXA-AS2 Facilitates Tumorigenesis and Progression of Papillary Thyroid Cancer by Modulating the miR-15a-5p/HOXA3 Axis. Hum Gene Ther. 2019; 30:618–631. 10.1089/hum.2018.109 30375256

[12] Jendrzejewski J, Thomas A, Liyanarachchi S, Eiterman A, Tomsic J, He H, Radomska HS, Li W, Nagy R, Sworczak K, de la Chapelle A. PTCSC3 is involved in papillary thyroid carcinoma development by modulating S100A4 gene expression. J Clin Endocrinol Metab. 2015; 100:E1370–77. 10.1210/jc.2015-2247 26274343 PMC4596031

[13] Goedert L, Plaça JR, Fuziwara CS, Machado MC, Plaça DR, Almeida PP, Sanches TP, Santos JF, Corveloni AC, Pereira IE, de Castro MM, Kimura ET, Silva WA Jr, Espreafico EM. Identification of long noncoding RNAs deregulated in papillary thyroid cancer and correlated with BRAF^V600E^ mutation by bioinformatics integrative analysis. Sci Rep. 2017; 7:1662. 10.1038/s41598-017-01957-0 28490781 PMC5431778

[14] Lytle JR, Yario TA, Steitz JA. Target mRNAs are repressed as efficiently by microRNA-binding sites in the 5’ UTR as in the 3’ UTR. Proc Natl Acad Sci USA. 2007; 104:9667–72. 10.1073/pnas.0703820104 17535905 PMC1887587

[15] Filipowicz W, Bhattacharyya SN, Sonenberg N. Mechanisms of post-transcriptional regulation by microRNAs: are the answers in sight? Nat Rev Genet. 2008; 9:102–14. 10.1038/nrg2290 18197166

[16] Bartel DP. MicroRNAs: target recognition and regulatory functions. Cell. 2009; 136:215–33. 10.1016/j.cell.2009.01.002 19167326 PMC3794896

[17] Hata A. Functions of microRNAs in cardiovascular biology and disease. Annu Rev Physiol. 2013; 75:69–93. 10.1146/annurev-physiol-030212-183737 23157557 PMC5215839

[18] Ceribelli A, Satoh M, Chan EK. MicroRNAs and autoimmunity. Curr Opin Immunol. 2012; 24:686–91. 10.1016/j.coi.2012.07.011 22902047 PMC3508200

[19] Lee JC, Gundara JS, Glover A, Serpell J, Sidhu SB. MicroRNA expression profiles in the management of papillary thyroid cancer. Oncologist. 2014; 19:1141–47. 10.1634/theoncologist.2014-0135 25323484 PMC4221366

[20] Aragon Han P, Weng CH, Khawaja HT, Nagarajan N, Schneider EB, Umbricht CB, Witwer KW, Zeiger MA. MicroRNA expression and association with clinicopathologic features in papillary thyroid cancer: a systematic review. Thyroid. 2015; 25:1322–29. 10.1089/thy.2015.0193 26414548

[21] Paraskevopoulou MD, Hatzigeorgiou AG. Analyzing MiRNA-LncRNA interactions. Methods Mol Biol. 2016; 1402:271–86. 10.1007/978-1-4939-3378-5_21 26721498

[22] Hatakeyama S. TRIM proteins and cancer. Nat Rev Cancer. 2011; 11:792–804. 10.1038/nrc3139 21979307

[23] Hatakeyama S. Early evidence for the role of TRIM29 in multiple cancer models. Expert Opin Ther Targets. 2016; 20:767–70. 10.1517/14728222.2016.1148687 26838517

[24] Wang L, Heidt DG, Lee CJ, Yang H, Logsdon CD, Zhang L, Fearon ER, Ljungman M, Simeone DM. Oncogenic function of ATDC in pancreatic cancer through Wnt pathway activation and beta-catenin stabilization. Cancer Cell. 2009; 15:207–19. 10.1016/j.ccr.2009.01.018 19249679 PMC2673547

[25] Xu J, Li Z, Su Q, Zhao J, Ma J. TRIM29 promotes progression of thyroid carcinoma via activating P13K/AKT signaling pathway. Oncol Rep. 2017; 37:1555–64. 10.3892/or.2017.5364 28098872

[26] Yuan Z, Villagra A, Peng L, Coppola D, Glozak M, Sotomayor EM, Chen J, Lane WS, Seto E. The ATDC (TRIM29) protein binds p53 and antagonizes p53-mediated functions. Mol Cell Biol. 2010; 30:3004–15. 10.1128/MCB.01023-09 20368352 PMC2876676

[27] Tang ZP, Dong QZ, Cui QZ, Papavassiliou P, Wang ED, Wang EH. Ataxia-telangiectasia group D complementing gene (ATDC) promotes lung cancer cell proliferation by activating NF-κB pathway. PLoS One. 2013; 8:e63676. 10.1371/journal.pone.0063676 23776433 PMC3680444

[28] Zou Z, Ma T, He X, Zhou J, Ma H, Xie M, Liu Y, Lu D, Di S, Zhang Z. Long intergenic non-coding RNA 00324 promotes gastric cancer cell proliferation via binding with HuR and stabilizing FAM83B expression. Cell Death Dis. 2018; 9:717. 10.1038/s41419-018-0758-8 29915327 PMC6006375

[29] Pan ZH, Guo XQ, Shan J, Luo SX. LINC00324 exerts tumor-promoting functions in lung adenocarcinoma via targeting miR-615-5p/AKT1 axis. Eur Rev Med Pharmacol Sci. 2018; 22:8333–42. 10.26355/eurrev_201812_16531 30556874

[30] Dong Y, Wan G, Yan P, Qian C, Li F, Peng G. Long noncoding RNA LINC00324 promotes retinoblastoma progression by acting as a competing endogenous RNA for microRNA-769-5p, thereby increasing STAT3 expression. Aging (Albany NY). 2020; 12:7729–46. 10.18632/aging.103075 32369777 PMC7244063

[31] Gao J, Dai C, Yu X, Yin XB, Zhou F. Long noncoding RNA LINC00324 exerts protumorigenic effects on liver cancer stem cells by upregulating fas ligand via PU box binding protein. FASEB J. 2020; 34:5800–17. 10.1096/fj.201902705RR 32128906

[32] Wu S, Gu Z, Wu Y, Wu W, Mao B, Zhao S. LINC00324 accelerates the proliferation and migration of osteosarcoma through regulating WDR66. J Cell Physiol. 2020; 235:339–48. 10.1002/jcp.28973 31225659

[33] Yin Y, Hong S, Yu S, Huang Y, Chen S, Liu Y, Zhang Q, Li Y, Xiao H. MiR-195 inhibits tumor growth and metastasis in papillary thyroid carcinoma cell lines by targeting CCND1 and FGF2. Int J Endocrinol. 2017; 2017:6180425. 10.1155/2017/6180425 28740507 PMC5504932

[34] Militello G, Weirick T, John D, Döring C, Dimmeler S, Uchida S. Screening and validation of lncRNAs and circRNAs as miRNA sponges. Brief Bioinform. 2017; 18:780–88. 10.1093/bib/bbw053 27373735

[35] Ni X, Xie JK, Wang H, Song HR. Knockdown of long non-coding RNA LINC00324 inhibits proliferation, migration and invasion of colorectal cancer cell via targeting miR-214-3p. Eur Rev Med Pharmacol Sci. 2019; 23:10740–50. 10.26355/eurrev_201912_19775 31858541

[36] Zhang M, Lin B, Liu Y, Huang T, Chen M, Lian D, Deng S, Zhuang C. LINC00324 affects non-small cell lung cancer cell proliferation and invasion through regulation of the miR-139-5p/IGF1R axis. Mol Cell Biochem. 2020; 473:193–202. 10.1007/s11010-020-03819-2 32734536

[37] Wan JF, Wan JY, Dong C, Li L. Linc00324 promotes the progression of papillary thyroid cancer via regulating Notch signaling pathway. Eur Rev Med Pharmacol Sci. 2020; 24:6818–24. 10.26355/eurrev_202006_21671 32633374

[38] Yu W, Liang X, Li X, Zhang Y, Sun Z, Liu Y, Wang J. MicroRNA-195: a review of its role in cancers. Onco Targets Ther. 2018; 11:7109–23. 10.2147/OTT.S183600 30410367 PMC6200091

[39] Lima CR, Gomes CC, Santos MF. Role of microRNAs in endocrine cancer metastasis. Mol Cell Endocrinol. 2017; 456:62–75. 10.1016/j.mce.2017.03.015 28322989

[40] Cong D, He M, Chen S, Liu X, Liu X, Sun H. Expression profiles of pivotal microRNAs and targets in thyroid papillary carcinoma: an analysis of the cancer genome atlas. Onco Targets Ther. 2015; 8:2271–77. 10.2147/OTT.S85753 26345235 PMC4556042

[41] Wang F, Jiang C, Sun Q, Yan F, Wang L, Fu Z, Liu T, Hu F. miR-195 is a key regulator of Raf1 in thyroid cancer. Onco Targets Ther. 2015; 8:3021–28. 10.2147/OTT.S90710 26527888 PMC4621222

[42] Yang R, Xing L, Zheng X, Sun Y, Wang X, Chen J. The circRNA circAGFG1 acts as a sponge of miR-195-5p to promote triple-negative breast cancer progression through regulating CCNE1 expression. Mol Cancer. 2019; 18:4. 10.1186/s12943-018-0933-7 30621700 PMC6325825

[43] Zhou S, Yu L, Xiong M, Dai G. LncRNA SNHG12 promotes tumorigenesis and metastasis in osteosarcoma by upregulating Notch2 by sponging miR-195-5p. Biochem Biophys Res Commun. 2018; 495:1822–32. 10.1016/j.bbrc.2017.12.047 29229388

[44] Yang C, Wu K, Wang S, Wei G. Long non-coding RNA XIST promotes osteosarcoma progression by targeting YAP via miR-195-5p. J Cell Biochem. 2018; 119:5646–56. 10.1002/jcb.26743 29384226

[45] Kong F, Ma J, Yang H, Yang D, Wang C, Ma X. Long non-coding RNA PVT1 promotes Malignancy in human endometrial carcinoma cells through negative regulation of miR-195-5p. Biochim Biophys Acta Mol Cell Res. 2018; 1865:1479–90. 10.1016/j.bbamcr.2018.07.008 30031900

